# Using FluoZin-3 and fura-2 to monitor acute accumulation of free intracellular Cd^2+^ in a pancreatic beta cell line

**DOI:** 10.1007/s10534-019-00226-z

**Published:** 2019-11-21

**Authors:** Latha M. Malaiyandi, Harsh Sharthiya, Ameir N. Barakat, Joshua R. Edwards, Kirk E. Dineley

**Affiliations:** Departments of Anatomy, College of Graduate Studies, Midwestern University, Downers Grove, IL 60515, USA; Departments of Pharmacology, College of Graduate Studies, Midwestern University, 555 31st Street, Downers Grove, IL 60515, USA

**Keywords:** MIN6, Cadmium, Leadmium Green, Voltage-gated calcium channel, Metal toxicity

## Abstract

The understanding of cellular Cd^2+^ accumulation and toxicity is hampered by a lack of fluorescent indicators selective for intracellular free Cd^2+^ ([Cd^2+^]_i_). In this study, we used depolarized MIN6 mouse pancreatic beta cells as a model for evaluating [Cd^2+^]_i_ detection with commercially available fluorescent probes, most of which have been traditionally used to visualize [Ca^2+^]_i_ and [Zn^2+^]_i_. We trialed a panel of 12 probes including fura-2, FluoZin-3, Leadmium Green, Rhod-5N, indo-1, Fluo-5N, and others. We found that the [Zn^2+^]_i_ probe FluoZin-3 and the traditional [Ca^2+^]_i_ probe fura-2 responded most consistently and robustly to [Cd^2+^]_i_ accumulation mediated by voltage-gated calcium channels. While selective detection of [Cd^2+^]_i_ by fura-2 required the omission of Ca^2+^ from extracellular buffers, FluoZin-3 responded to [Cd^2+^]_i_ similarly in the presence or absence of extracellular Ca^2+^. Furthermore, we showed that FluoZin-3 and fura-2 can be used together for simultaneous monitoring of [Ca^2+^]_i_ and [Cd^2+^]_i_ in the same cells. None of the other fluorophores tested were effective [Cd^2+^]_i_ detectors in this model.

## Introduction

Cadmium is a highly toxic trace metal with virtually no biological function. Human exposure to cadmium occurs via cigarette smoking, ingestion of contaminated foodstuffs, and occupational and environmental exposure (Nordberg 2009). Depending on the route of intake and the timeframe over which it occurs, cadmium can be particularly toxic to kidney, liver, GI, bone, lung, and reproductive tissues. Some data suggest that cadmium may also be associated with pancreatic cancer and type II diabetes (Muayed et al. 2012; Edwards and Ackerman 2016; Djordjevic et al. 2019). Accordingly, the U.S. Agency for Toxic Substances and Disease Registry currently ranks cadmium 7th out of 275 environmental hazardous substances (ATSDR 2019). At the cellular level, Cd^2+^ toxicity is only partly understood. Intracellular accumulation can occur through a variety of pathways normally used by Ca^2+^, Zn^2+^, and other metals (Thévenod 2010; Bouron et al. 2014). Once inside the cell, the deleterious effects of Cd^2+^ probably result from its tendency to displace other biologically important metals from critical proteins, in a process that is referred to as ionic mimicry (Bridges and Zalups 2005).

One major limitation to our understanding of cadmium toxicity is a lack of fluorophores that are selective for intracellular free Cd^2+^ ([Cd^2+^]_i_) and are easy to use. The first live cell studies of [Cd^2+^]_i_ uptake appropriated the iconic Ca^2+^ probe fura-2, which also has extremely high affinity for Cd^2+^ (Hinkle et al. 1992). Those experiments were often performed in Ca^2+^-free solutions in order to minimize any Ca^2+^ contribution to the intracellular signal. More recently, the Cd^2+^- and Pb^2+^-sensitive probe Leadmium Green has aided the study of [Cd^2+^]_i_ handling in a variety of organisms including plants (Zanella et al. 2015; Greger et al. 2016), mammals (Romero et al. 2009; Miura et al. 2013), and microbes (Manzano et al. 2013; Johnson et al. 2018, 2019). The high-affinity Zn^2+^ sensor FluoZin-3 is also responsive to Cd^2+^ (Zhao et al. 2009), and this promiscuity has been exploited to monitor [Cd^2+^]_i_ uptake in marine invertebrates (Ortega et al. 2017a, b) and rodent hippocampal slices (Kay and Rumschik 2011). Several other small chemical ion sensors have demonstrated Cd^2+^ sensitivity in cell-free conditions, including FluoZin-1 (Lee et al. 2005), Rhod-5N (Soibinet et al. 2008), indo-1(Vo-Dinh et al. 1994), fura-2FF, and mag-fura-2 (Hyrc et al. 2000), but there is little or no data regarding their value in live-cell Cd^2+^ applications. Various laboratories have invented chemical and protein-based probes intended primarily for the detection of Cd^2+^, several of which demonstrated potential to selectively report Cd^2+^ amidst Zn^2+^ and other competing metals, but none of these prototypes has yet been adopted into common use for the live-cell imaging of [Cd^2+^]_i_ (Vinkenborg et al. 2011; Chiu et al. 2013; Taki 2013).

In this study we evaluated a panel of fluorescent probes for their ability to monitor [Cd^2+^]_i_ in a model of acute accumulation. We limited our consideration to fluorophores that are commercially available, because they are easily procured by labs with modest resources, and their properties tend to be fairly well documented in the literature. [Cd^2+^]_i_ uptake was assayed in depolarized mouse insulinoma cells (MIN6), which express much of the machinery important to pancreatic beta cell function, including L-type voltage-gated Ca^2+^ channels that are readily activated with high extracellular potassium (Zhou et al. 2014). Of the 12 probes we tested, the high affinity Zn^2+^ indicator FluoZin-3 and the classic Ca^2+^ probe fura-2 proved most useful as [Cd^2+^]_i_ detectors. We then conducted a series of experiments that compared the strengths and weaknesses of these two probes, including a dual dye method that uses both for the simultaneous monitoring of [Cd^2+^]_i_ and [Ca^2+^]_i_ inside the same cells.

## Materials and methods

### Reagents

Cell culture media, N,N,N′,N′-tetrakis (2-pyridylmethyl) ethylenediamine (TPEN), and fluorescent probes (Calcium Green-5N, Fluo-5N, FluoZin-1, FluoZin-2, FluoZin-3, fura-2, indo-1, Leadmium Green, mag-fura-2, Newport Green DCF, and Rhod-5N) were purchased from Thermo Fisher Scientific. Fura-2FF was purchased from TEF Labs. Fetal bovine serum was purchased from Midwest Scientific. All other reagents were purchased from Sigma-Aldrich unless otherwise noted.

### Cell culture

The mouse insulinoma cell line (MIN6) was generously provided by Dr. Malek El Muayed at Northwestern University, Feinberg School of Medicine and maintained according to previously published methods (Muayed et al. 2012). Briefly, cells were grown in high glucose Dulbecco’s Modified Eagle’s Medium (DMEM, Gibco #12430–054) supplemented with 0.1% 2-mercaptoethanol, 15% heat-inactivated fetal bovine serum and 1% penicillin/streptomycin (Sigma #P4333) at 37 °C and 5% CO_2_. Round glass coverslips (18 mm, Warner Instruments) were coated with poly-l-lysine (Sigma #P4707) according to the manufacturer’s recommended protocol, and trypsinized cells were plated at a density of 100,000 cells per coverslip placed in 12-well culture plates. Media was changed every 2 days, and cells were used 5–7 days following plating. Cells from passages 23–42 were used in experiments.

### Epifluorescence microscopy

Live-cell fluorescence microscopy was carried out in a HEPES-buffered, phosphate-free salt solution (HBSS) containing (in mM): 137 NaCl, 5 KCl, 10 NaHCO_3_, 0.9 MgSO_4_, 1.4 CaCl_2_, 20 HEPES, and 5.5 glucose, pH adjusted to 7.4 with NaOH. Depolarization was achieved with a high KCl, phosphate-free buffer containing (in mM): 92 NaCl, 50 KCl, 10 NaHCO_3_, 0.9 MgSO_4_, 1.4 CaCl_2_, 20 HEPES, and 5.5 glucose, pH adjusted to 7.4 with NaOH. For nominally Ca^2+^- free experiments, CaCl_2_ was omitted from both the HBSS and high KCl buffer. For loading of dye, cells were incubated for 30 min at room temperature in 500 μL loading buffer comprised of HBSS supplemented with 5 μM acetoxymethyl (AM) form of the fluorescent probe and bovine serum albumin (5 mg/mL). Coverslips with dye-loaded cells were placed in a 500 μL perfusion chamber and mounted on the microscope stage. The chamber was superfused at 5 mL/min with CaCl_2_, CdCl_2_, ZnSO_4_, sodium pyrithione, MnCl_2_, TPEN, and/or Ca^2+^ channel blockers at the indicated concentrations.

Live-cell microscopy of single dye experiments were performed at room temperature on an IX70 Olympus fluorescence microscope equipped with a 40× oil-immersion objective (UAPO/340, ND = 1.35). Excitation light was produced by a xenon lamp-based Lambda LS stand-alone illuminator with filter wheels (Sutter Instruments). Optics for fura-2, fura-2FF and mag-fura-2 used alternately switched 340/20 and 380/11 nm excitation filters, 400 nm dichromatic mirror, and 510/84 nm emission filter. Optics for indo-1 used 340/10 nm excitation filter, 387 nm dichromatic mirror, and alternately switched 405/30 and 485/25 nm emission filters. For Calcium Green-5N, Fluo-5N, FluoZin-1, FluoZin-2, FluoZin-3, Leadmium Green, and Newport Green DCF: 480/20 nm excitation filter; 500 nm dichromatic mirror; and 520 nm emission filter. For Rhod-5N: 540/20 nm excitation filter; 570 nm dichromatic mirror, and 590 nm emission filter. Images were captured with an LT Flash4.0 CMOS camera with HCImage acquisition software (Hamamatsu Photonics).

Dual dye experiments using fura-2 and FluoZin-3 were performed on a Nikon Eclipse TE2000-S fluorescence microscope equipped with a 40× oil-immersion objective (S FLUOR, ND = 1.30), illuminated by a xenon lamp-based Lambda DG-4 high speed filter changer (Sutter Instruments). Cells were incubated in loading buffer supplemented with 5 μM fura-2 and FluoZin-3 for 45 min at room temperature. For fura-2, excitation light was modified by alternately switched 340/11 and 380/11 nm excitation filters. For FluoZin-3, light was modified by a 495/10 nm excitation filter. A 510 nm dichromatic mirror and 560/80 emission filter were used for both dyes. The camera and software were the same types used in the Olympus system described above.

### Spectrofluorimetry

Spectrofluorimetric measurements of the tetrapotassium salt form of FluoZin-3 were performed on a Perkin Elmer Enspire multimode plate reader. Fluorescence was recorded from 96-well black plates with 495 nm excitation and 517 nm emission. Experiments were performed in Chelex 100-treated buffer containing (in mM): 140 KCl, 10 MOPS, 10 nitrilotriacetic acid (NTA), pH 7. FluoZin-3 salt was added at a final concentration of 500 nM, and serial dilutions of metal from 1000 × stock solutions of CdCl_2_ or ZnSO_4_ were added in the range of 0–9 mM, to a final well volume of 100 μL. Free metal concentrations were calculated using the Maxchelator program (https://somapp.ucdmc.ucdavis.edu/pharmacology/bers/maxchelator/webmaxc/webmaxcS.htm). Data were represented as a percentage of fluorescence at baseline (50 μM TPEN) versus log of free metal (in M). K_D_ values were calculated by curve-fitting the data using nonlinear regression of one-site specific binding with B_max_ constrained between 0 and 100.

### Inductively coupled plasma mass spectrometry (ICP-MS)

Total intracellular Cd^2+^ levels were measured by inductively coupled plasma mass spectrometry (ICP-MS) in samples sent to the Michigan State University Veterinary Diagnostic Laboratory. MIN6 cells were plated in 12-well plates coated with poly-l-lysine and maintained in growth media for 5–7 days. All HBSS buffers were made nominally Ca^2+^-free. Cells were rinsed twice in non-depolarizing HBSS, then treated with 0, 30 or 300 μM CdCl_2_ in 2 mL volume of depolarizing HBSS for 5 minutes. Other wells were treated with 30 μM CdCl_2_ in non-depolarizing HBSS. Plates were placed on a rocker during the 5-minute treatment to simulate bath perfusion use in the microscopy experiments. Cells were rinsed three times in non-depolarizing HBSS containing 100 μM EDTA to remove any extracellular bound Cd^2+^, followed by a final rinse in non-depolarizing HBSS. Cells were lysed using 0.1 mL commercially available lysing buffer (Thermo Fisher Scientific 78505). For each trial, cell lysates were pooled from three wells and experiments were repeated in at least three different cell culture passages.

### Statistical analysis

Statistical analyses and graphical representations of data were performed with GraphPad Prism 8.1 software. For the fura dyes, the ratio was calculated as the background-subtracted signal at 340 nm divided by the background-subtracted signal at 380 nm. For indo-1, the ratio was similarly calculated as the background-subtracted signal at 405 nm divided by the background subtracted signal at 485 nm. For single wavelength dyes, fluorescence signal (F) was divided by the starting fluorescence value (F_0_), after background-subtraction. For a given coverslip, fluorescence was typically collected from a field of 30–60 cells. Representative data traces are plotted as an averaged fluorescence change from all cells within a field from one coverslip. Summary data are presented in bar graphs as mean ± SD consisting of four to six coverslips for each condition, using cells from at least three different cell culture preparations. Where appropriate, statistical analyses used Welch’s t-test or ANOVA with Tukey’s post hoc test for multiple comparisons. Statistical significance was determined as p ≤ 0.05.

## Results

**Fig. 1:**
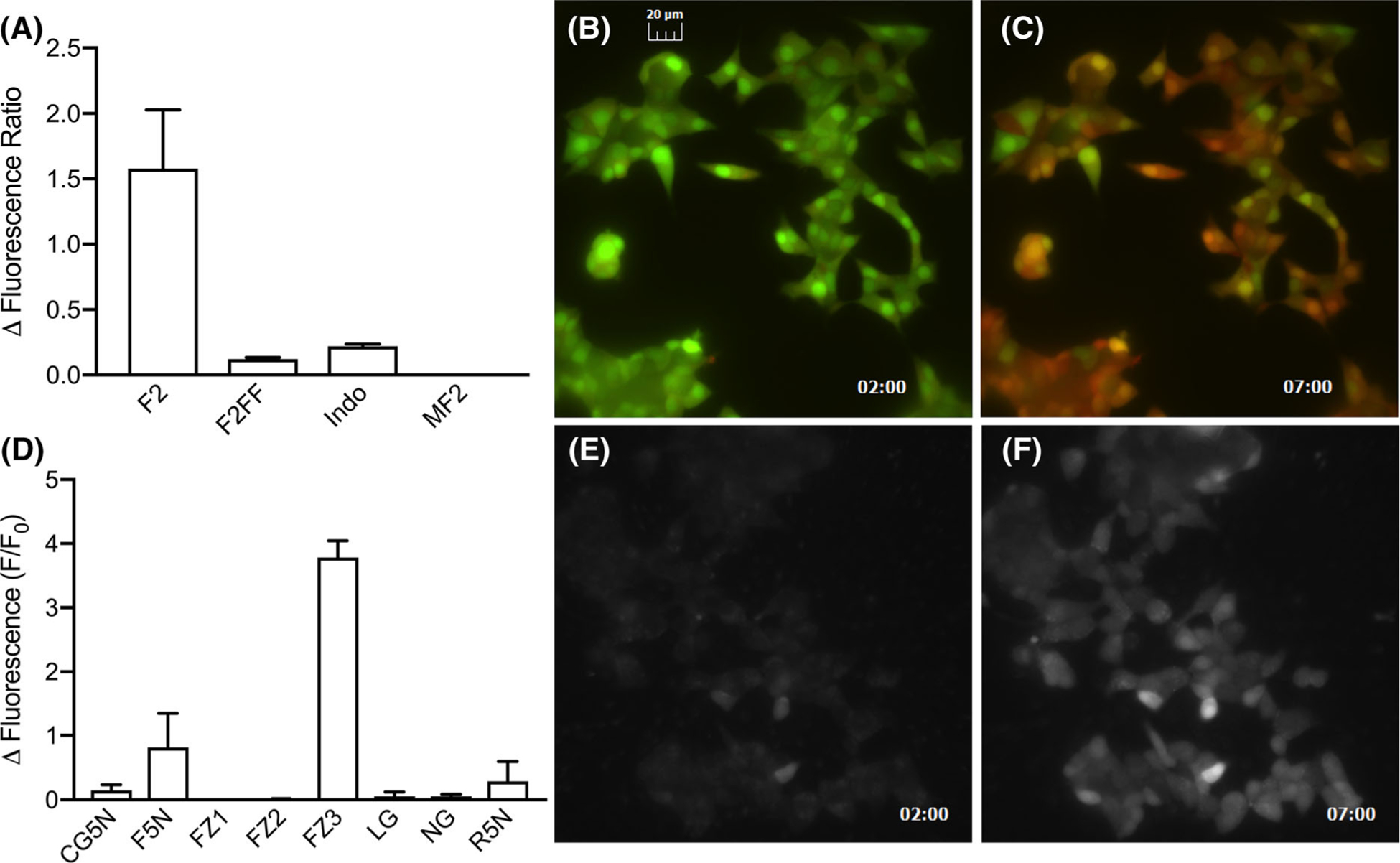
Summary of ratiometric (**a**) or single wavelength (**d**) dye responses to 5-min depolarizing stimulus supplemented with CdCl_2_ (30 μM). Bars represent mean ± SD change in (**a**) fluorescence ratio or (**d**) F/F_0_. These trials were carried out in nominally Ca^2+^-free buffer. Representative fluorescence micrographs before and after 30 μM CdCl_2_ and depolarization, for fura-2 (**b**, **c**) or FluoZin-3 (**e**, **f**). *F2* fura-2, *F2FF* Fura-2FF, *Indo* Indo-1, *MF2* mag-fura-2, *F5N* Fluo-5N, *FZ1* FluoZin-1, *FZ2* FluoZin-2, *FZ3* FluoZin-3, *LG* Leadmium Green, *NG* Newport Green DCF, *R5N* Rhod-5N, *CG5N* Calcium Green-5N. Time stamp is in minute:second format

Our standard stimulus for provoking [Cd^2+^]_i_ accumulation in MIN6 cells used HBSS buffer containing 50 mM KCl and supplemented with 30 μM CdCl_2_, applied for 5 minutes. This triggers rapid depolarization, activation of voltage-gated Ca^2+^ channels, and Cd^2+^ influx. Of the assortment of small molecule ratiometric and single wavelength probes that are potentially useful as live-cell Cd^2+^ sensors, only two that we tested, fura-2 and FluoZin-3, responded to [Cd^2+^]_i_ accumulation in consistent and robust fashion (mean fura-2 ratio change = 1.58; mean FluoZin-3 F/F_0_ change = 3.78; Fig. 1). The fluorescence micrographs show dye signal that is for the most part evenly distributed throughout the cell, indicating minimal compartmentalization of metal or probe. All of our subsequent experiments focused on fura-2 and Fluo-Zin-3.

**Fig. 2:**
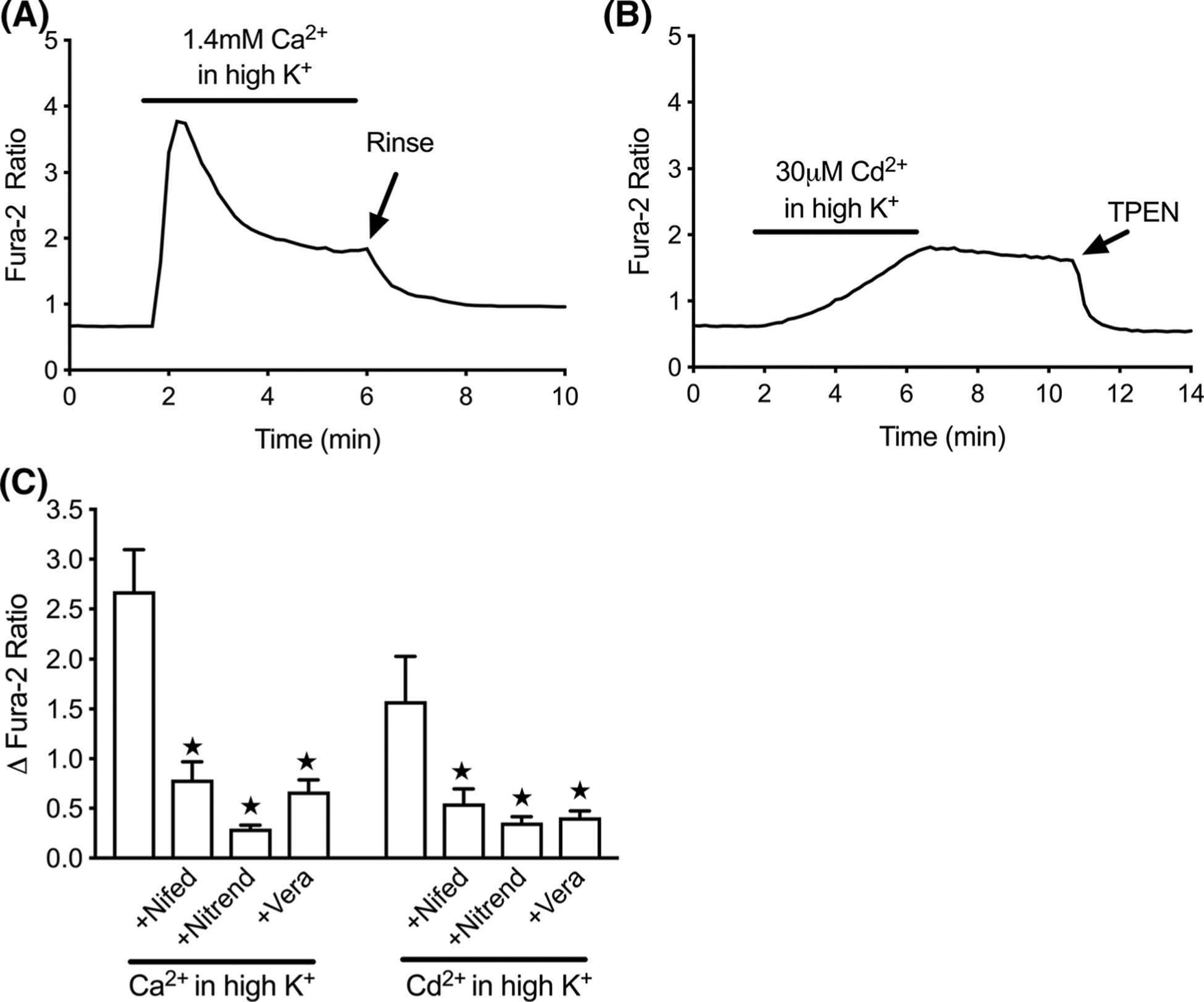
Comparison of fura-2 detection of depolarization-induced [Ca^2+^]_i_ and [Cd^2+^]_i_ accumulation. **a** Representative fura-2 response to depolarization for 5 min in the presence of CaCl_2_ (1.4 mM), followed by HBSS rinse. **b** Representative fura-2 response to depolarization for 5 min in the presence of CdCl_2_ (30 μM), followed by 5 min rinse, then TPEN (50 μM). **c** Summary data for VGCC inhibition of fura-2 response to depolarization-induced accumulation of [Ca^2+^]_i_ or [Cd^2+^]_i_. For experiments with VGCC blockers, cells were pretreated for 3 min with 1 μM nifedipine, nitrendipine, or verapamil prior to depolarization stimulus. All Cd^2+^ trials were performed in nominally Ca^2+^-free buffer. Bars represent mean ± SD fura-2 ratio change. ★p < 0.05 using Welch’s t-test

**Fig. 3:**
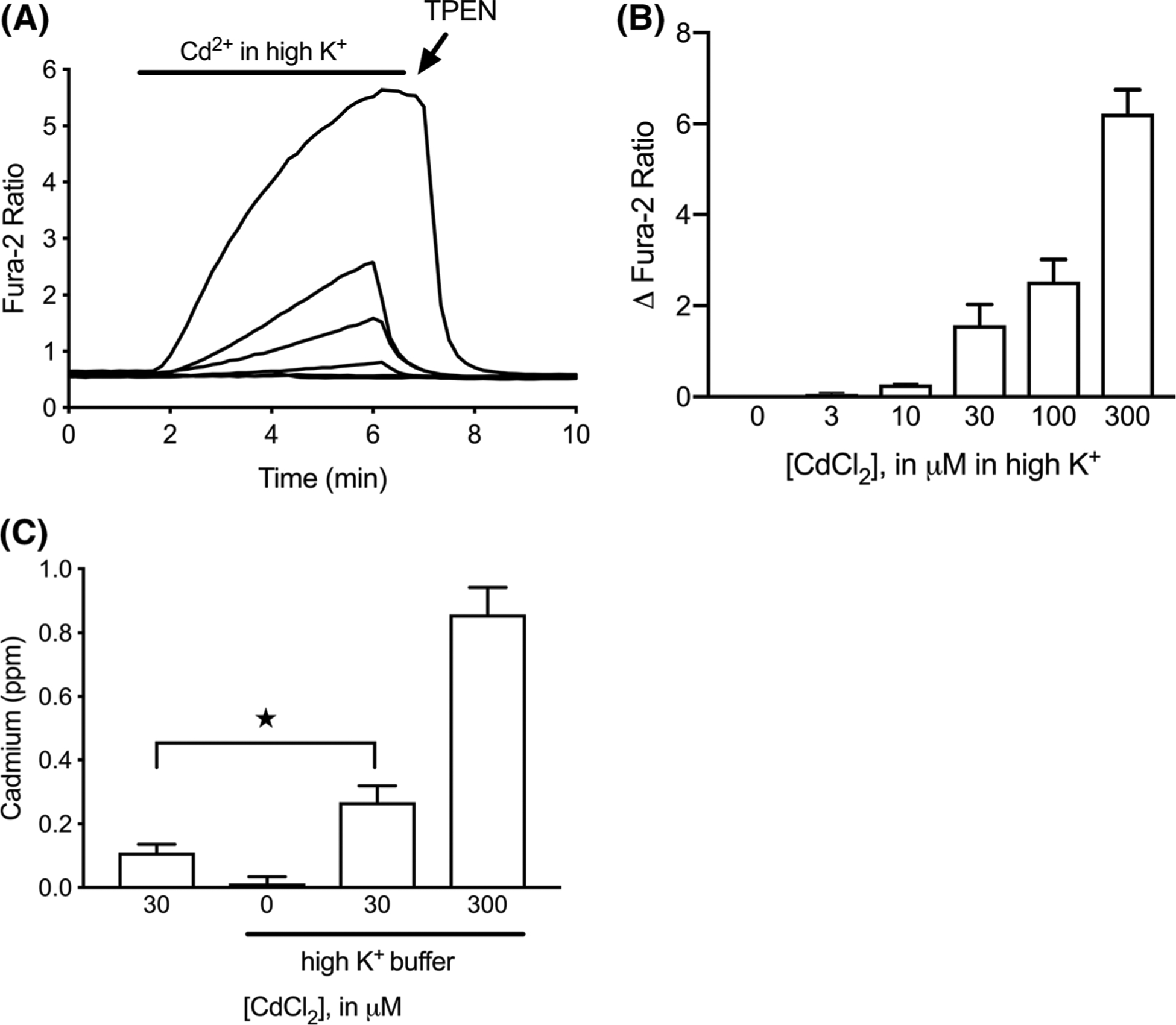
Fura-2 concentration-dependent response to depolarization-induced [Cd^2+^]_i_ accumulation. **a** Representative traces from cells depolarized for 5 min in the presence of CdCl_2_ (0, 3, 10, 30, 100, and 300 μM), then treated with 50 μM TPEN. **b** Summary of peak fura-2 ratio changes from experiments in **a**. **c** ICP-MS measurements of Cd^2+^ accumulation in MIN6 cells. Bars represent mean ± SD ratio change. These trials were carried out in nominally Ca^2+^-free buffer. ★p < 0.05 using one-way ANOVA with Tukey’s multiple comparisons test

We next examined more carefully the utility of fura-2 in our model by comparing the signal characteristics associated with [Cd^2+^]_i_ and [Ca^2+^]_i_ accumulation. Figure 2a shows a typical fura-2 depolarization response in an excitable cell, under conditions where extracellular Ca^2+^ is abundant. Notable features included a fast, robust rise in signal that declined quickly to a secondary plateau, which then decayed slowly over the duration of the depolarizing stimulus. Termination of depolarization was sufficient to permit the signal to return quickly and nearly completely to starting values. Figure 2b shows the fura-2 response to [Cd^2+^]_i_ accumulation (30 μM extracellular Cd^2+^) when Ca^2+^ is omitted from the buffer. In contrast to the kinetics of the [Ca^2+^]_i_ signal, the [Cd^2+^]_i_ rise was much more gradual and remained elevated after depolarization was finished. Restoration of baseline fluorescence did not occur spontaneously but instead required application of the membrane-permeant, heavy metal chelator TPEN (50 μM). We confirmed that L-type voltage-gated Ca^2+^ channels (VGCCs) are a major route of accumulation for both ions under these circumstances. The standard L-type blockers nifedipine, nitrendipine, and verapamil inhibited fura-2 responses as much as 89% for [Ca^2+^]_i_ and 77% for [Cd^2+^]_i_ (Fig. 2c). The fura-2 response to [Cd^2+^]_i_ proved to be concentration-dependent across values tested up to 300 μM extracellular Cd^2+^, and 10 μM extracellular Cd^2+^ was the apparent lower limit for delivering enough intracellular Cd^2+^ to produce a perceptible fura-2 response (Figs. 3a, b). These results were supported by ICP-MS measurements of whole cell cadmium (Fig. 3c), which show that depolarization increased cellular cadmium content, and that intracellular cadmium accumulation was broadly proportional to extracellular Cd^2+^.

**Fig. 4:**
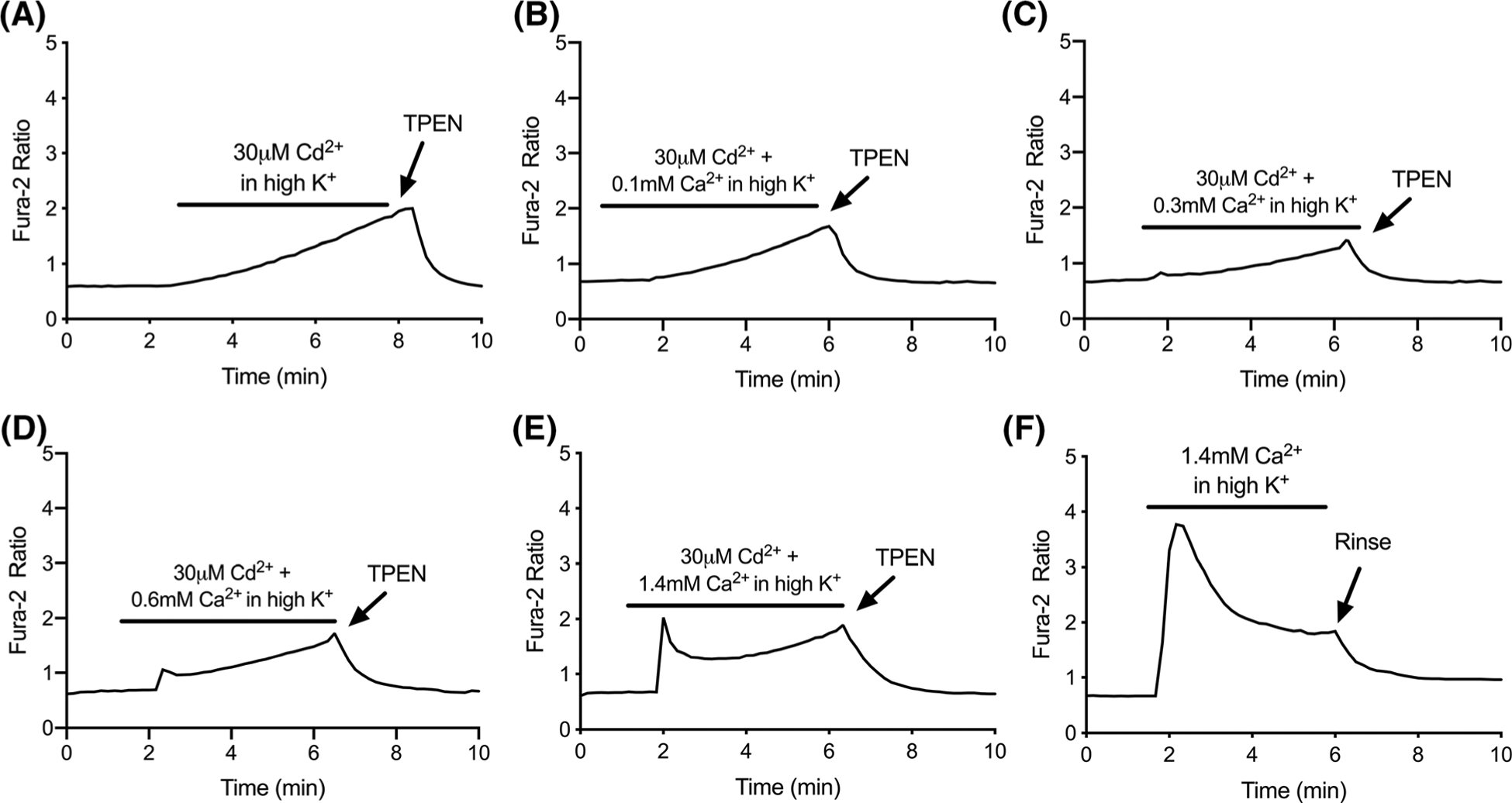
Representative traces of fura-2 response to depolarization-induced [Cd^2+^]_i_ accumulation in the presence of extracellular Ca^2+^. **a**–**e** Cells were depolarized for 5 min in the presence of CdCl_2_ (30 μM), and varying extracellular CaCl_2_ (0, 0.1, 0.3, 0.6, and 1.4 mM). **f** For comparison, representative trace of fura-2 response to CaCl_2_ (1.4 mM) in depolarizing conditions without CdCl_2_

Up to this point, the [Cd^2+^]_i_ trials were all performed in the absence of any added Ca^2+^ (i.e. nominally Ca^2+^-free buffer). While this tactic helps to minimize signal confounds that are expected when exploiting a high-affinity [Ca^2+^]_i_ probe for the detection of [Cd^2+^]_i_, it diminishes the physiological relevance of these experiments. In Fig. 4, we explored how fura-2 detection of [Cd^2+^]_i_ changes over a range of extracellular Ca^2+^ concentrations. When extracellular Ca^2+^ is absent or minimal (Fig. 4a–c), the fura-2 response is dominated by [Cd^2+^]_i_, as shown by the slow and continuous rise, and TPEN reversal. At 0.6 mM extracellular Ca^2+^ (Fig. 4d), the signal became embellished with a swift rise that began immediately upon depolarization, which was further enhanced when extracellular Ca^2+^ was restored to physiologic levels (1.4 mM, Fig. 4e). The rapid initial response was followed by a secondary phase where the signal climbed slowly but continuously for the duration of the depolarizing stimulus. Presumably, this secondary rise reflects the gradual accumulation of [Cd^2+^]_i_ in cells that already have elevated [Ca^2+^]_i_. Thus, the trace in Fig. 4e can be viewed as a hybrid product of the traces obtained in Fig. 4a and 4f with Cd^2+^or Ca^2+^ alone, respectively.

**Fig. 5:**
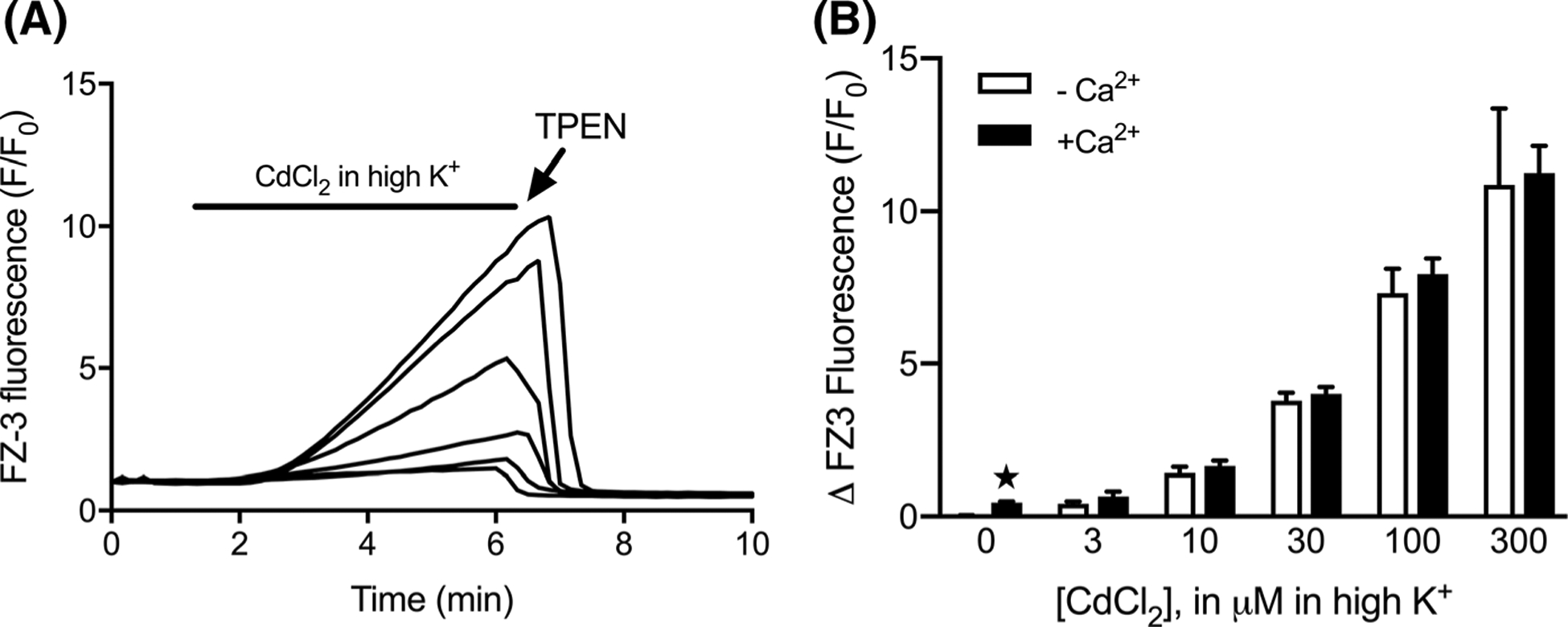
FluoZin-3 response to depolarization-induced [Cd^2+^]_i_ accumulation amidst physiological extracellular Ca^2+^. **a** Representative traces of cells depolarized for 5 min in the presence of CdCl_2_ (0, 3, 10, 30, 100 and 300 μM) and 1.4 mM CaCl_2_, then treated with 50 μM TPEN. **b** Comparison of peak fluorescence induced by [Cd^2+^]_i_ in the presence (solid) and absence (open) of 1.4 mM extracellular Ca^2+^. Bars represent mean ± SD FluoZin-3 F/F_0_ change. ★p < 0.05 using Welch’s t-test

**Fig. 6:**
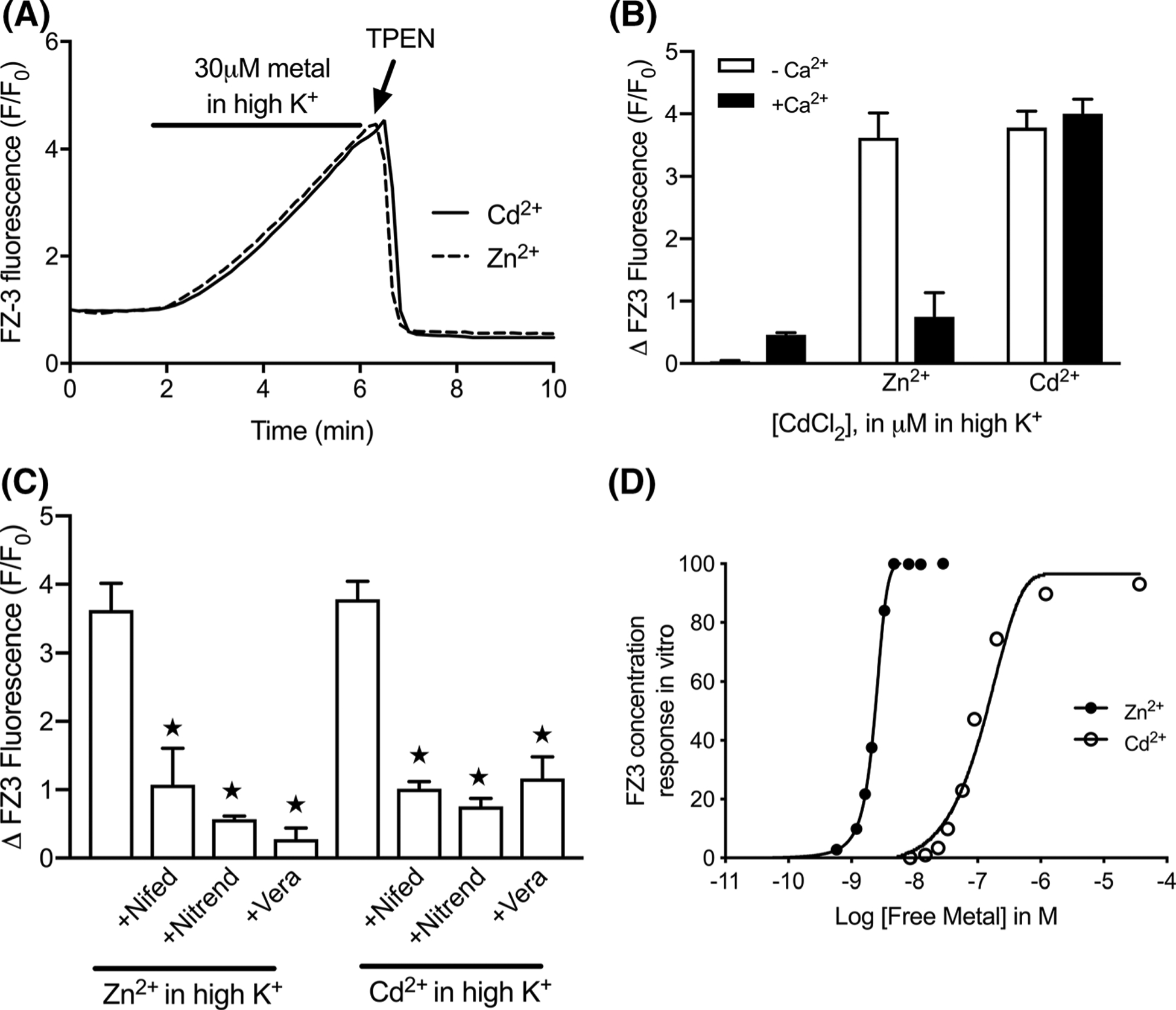
Comparison of FluoZin-3 detection of depolarization-induced accumulation of [Cd^2+^]_i_ and [Zn^2+^]_i_. **a** Representative traces of cells depolarized for 5 min in the presence of 30 μM CdCl_2_ (solid line) or 30 μM ZnSO_4_ (dashed line), then treated with 50 μM TPEN. **b** Peak fluorescence induced by [Zn^2+^]_i_ or [Cd^2+^]_i_ in the absence (open bars) or presence (solid bars) of 1.4 mM extracellular CaCl_2_ in depolarizing conditions. **c** Summary data for VGCC inhibition of FluoZin-3 changes in response to depolarization-induced [Zn^2+^]_i_ or [Cd^2+^]_i_ accumulation. For experiments with VGCC blockers, cells were pretreated for 3 min with 1 μM nifedipine, nitrendipine, or verapamil prior to depolarization stimulus. Trials in **a**, **c** were carried out in nominally Ca^2+^-free buffer. Bars represent mean ± SD FluoZin-3 F/F_0_ change; ★p < 0.05 using Welch’s t-test. **d** In vitro comparisons of FluoZin-3 response to Cd^2+^ (open) and Zn^2+^ (solid). FluoZin-3 fluorescence was measured in the presence of free Cd^2+^ (0–3.69 × 10^−5^ M; open) or Zn^2+^ (0–2.82 × 10^−8^ M; solid). Fluorescence was expressed as percent of maximal fluorescence achieved with Zn^2+^. Calculated dissociation constants, K_D_, for Zn^2+^ and Cd^2+^ are 3.67 nM and 129 nM, respectively

These data demonstrate that fura-2 can be used to monitor [Cd^2+^]_i_ with reasonable sensitivity, provided that extracellular Ca^2+^ is scarce. Yet even in the presence of physiological extracellular Ca^2+^, the component contribution of each ion can be perceived in the fura-2 recording. Still, because it is not possible to precisely dissect the two signals, the practical use of fura-2 in this context is limited. FluoZin-3 is a single wavelength Zn^2+^ indicator, that is insensitive to Ca^2+^, but showed promise as an [Cd^2+^]_i_ sensor in our pilot assays (Fig. 1). Throughout a series of depolarization trials in 1.4 mM CaCl_2_, FluoZin-3 responded to [Cd^2+^]_i_ in concentration-dependent fashion, with F/F_0_ increasing by roughly 11-fold (Fig. 5a, b). The gradual rise of FluoZin-3 was kinetically consistent with the [Cd^2+^]_i_-induced fura-2 changes observed in Fig. 3a. In contrast to fura-2 however, the presence or absence of extracellular Ca^2+^ did not alter the FluoZin-3 response to the Cd^2+^ challenge, suggesting that [Ca^2+^]_i_ accumulation does not meaningfully confound the FluoZin-3 response to [Cd^2+^]_i_. We noted that Ca^2+^ influx alone (i.e. depolarization in the presence of 1.4 mM CaCl_2_, without added Cd^2+^) stimulated a small but consistent FluoZin-3 increase (about 1.5-fold). This was prevented by co-treatment with TPEN (data not shown), suggesting that Ca^2+^ influx mobilized a small amount of intracellular Zn^2+^, which is consistent with previous reports that documented Ca^2+^-induced Zn^2+^ mobilization in other cell types (Dineley et al. 2008; Li et al. 2011; Duncan et al. 2016). For comparative purposes, we also conducted FluoZin-3 trials with depolarization-induced Zn^2+^- influx (Fig. 6). In nominally Ca^2+^-free conditions, the signal elicited by 30 μM Zn^2+^ was similar to that obtained with 30 μM Cd^2+^ (Fig. 6a, b). In contrast to [Cd^2+^]_i_ however, FluoZin-3 reporting of [Zn^2+^]_i_ was markedly reduced in the presence of 1.4 mM CaCl_2_. This suggests that Zn^2+^, unlike Cd^2+^, is a rather poor competitor for Ca^2+^ permeable pathways, under depolarizing circumstances when Ca^2+^ is abundant. The FluoZin-3 responses were also reduced by approximately 80% for Zn^2+^ and 92% for Cd^2+^ with VGCC blockers (Fig. 6c). It is important to note that equivalent intracellular fluorescence does not necessarily indicate equivalent amounts of intracellular metal. Determination of FluoZin-3 affinities yielded dissociation constants of 3.67 nM for Zn^2+^, and 129 nM for Cd^2+^ (Fig. 6d)—values that are broadly consistent with previous publications. This difference in affinities and also, possibly, in quantum yields and other factors means that a much smaller concentration of Zn-FluoZin-3 could potentially produce the same fluorescence intensity as a much larger amount of Cd-FluoZin-3.

**Fig. 7:**
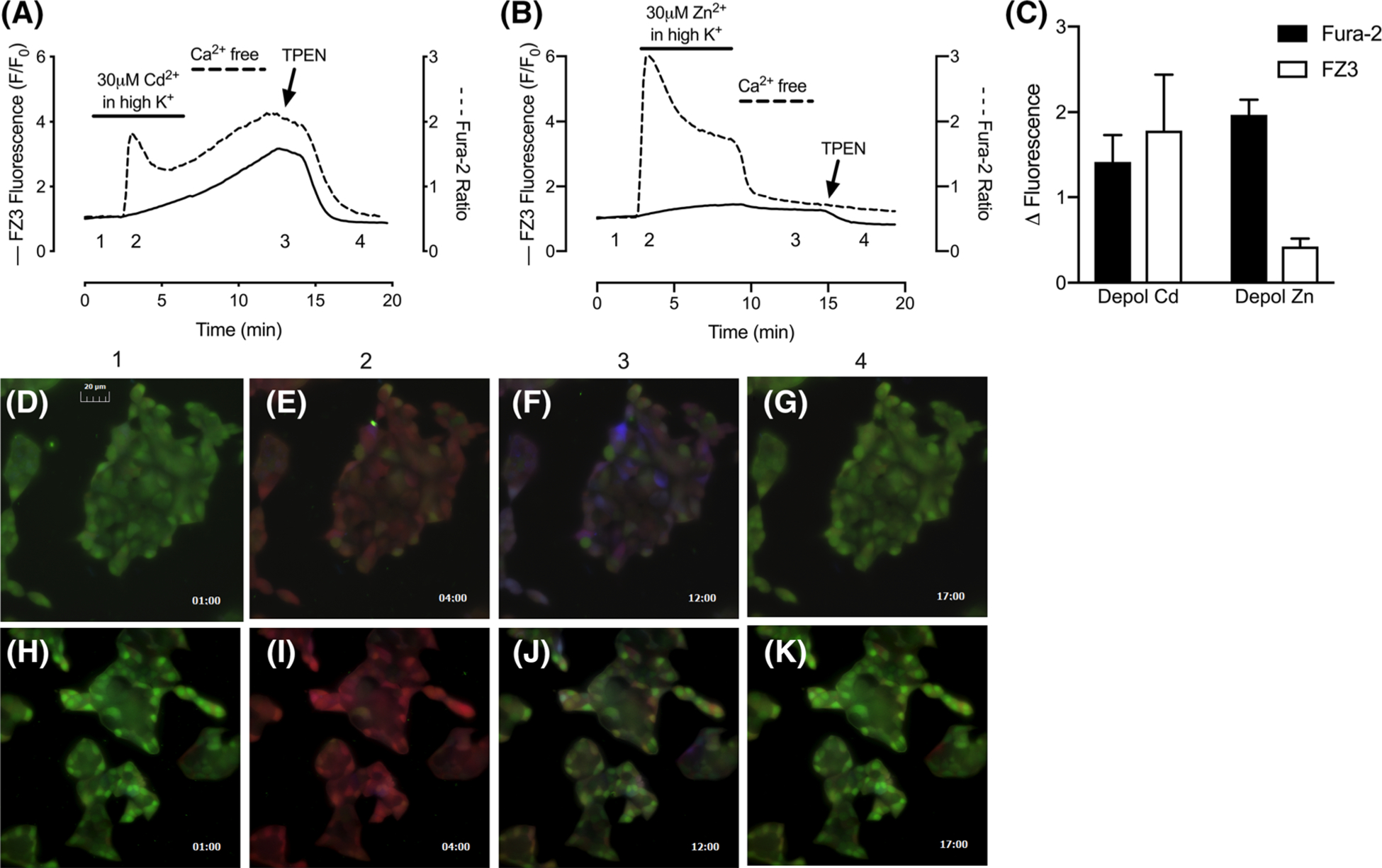
Simultaneous monitoring of [Cd^2+^]_i_ and [Ca^2+^]_i_, or [Zn^2+^] and [Ca^2+^]_i_ with dual dye approach. **a** Representative fura-2 and FluoZin-3 traces recorded from cells co-loaded with both dyes and depolarized for 5 min in the presence of 1.4 mM CaCl_2_ and 30 μM CdCl_2_, rinsed with Ca^2+^-free buffer for 5 min, then treated with 50 μM TPEN. Left axis corresponds to FluoZin-3 F/F_0_ (solid line); right axis corresponds to fura-2 ratio (dotted line). **b** Same paradigm as in **a**, but with 30 μM ZnSO_4_ instead of CdCl_2_. **c** Summary changes (mean ± SD) in fura-2 ratio (solid bars) or FluoZin-3 F/F_0_ (open bars) in response to Cd^2+^ or Zn^2+^ under depolarizing conditions. **d**–**k**. Composite fluorescence micrographs of fura-2 and FluoZin-3 in response to [Cd^2+^]_i_ and [Ca^2+^]_i_ (**d**–**g**), or [Zn^2+^]_i_ and [Ca^2+^]_i_ (**h**–**k**). Micrographs correspond to timepoints 1–4 indicated on the representative traces: 1 (**d**, **h**), 2 (**e**, **i**), 3 (**f**, **j**) and 4 (**g**, **k**). Time stamp in minute:second format

The spectral compatibility of fura-type probes with FluoZin-3 allows both dyes to be surveyed simultaneously with basic live-cell imaging equipment (Devinney et al. 2005). This, combined with the facts that FluoZin-3 responds well to Cd^2+^ but is insensitive to Ca^2+^, and that fura-2 responds very well to Ca^2+^, suggested that both dyes could be used together for concurrent detection of [Cd^2+^]_i_ and [Ca^2+^]_i_. Figure 7a shows a dual dye approach to simultaneous monitoring of intracellular Cd^2+^ and Ca^2+^ in depolarized MIN6 cells. The results are approximately similar to those obtained by using the dyes individually, however the FluoZin-3 signal is somewhat suppressed, compared to the analogous 30 μM Cd^2+^ trial in Fig. 5. This is surely due to some fraction of the [Cd^2+^]_i_ load interacting with fura-2 instead of FluoZin-3. Nevertheless, a robust and consistent FluoZin-3 response was obtained. Because these same principles can be applied to the simultaneous detection of [Zn^2+^]_i_ and [Ca^2+^]_i_, we then tested the dual dye approach with 30 μM ZnSO_4_ and 1.4 mM CaCl_2_, under depolarizing conditions. In this paradigm, FluoZin-3 yielded a paltry response, while the fura-2 response was rapid and robust, which is consistent with the limited [Zn^2+^]_i_ accumulation observed with FluoZin-3, in the presence of extracellular Ca^2+^, in Fig. 5b. Another important observation reinforced by these trials is the lack of fura-2 recovery associated with [Cd^2+^]_i_ accumulation. This contrasts with the [Zn^2+^]_i_ trials, where a short rinse was sufficient to return the fura-2 signal to pre-stimulus values. However, under circumstances of [Cd^2+^]_i_ accumulation, both dye signals remained elevated, until TPEN was applied. The impact of [Cd^2+^]_i_ on intracellular transport processes is largely unknown, however it is reasonable to assume that elevated [Ca^2+^]_i_ persists because [Cd^2+^]_i_ disrupts [Ca^2+^]_i_ clearance mechanisms (Biagioli et al. 2008). Fluorescence micrographs in Fig. 7d–k present pseudocolorized, three-part composite images that assign the 340 nm fura-2 signal to the red channel, 380 nm fura-2 signal to the green channel, and 490 nm FluoZin-3 signal to the blue channel. The images were taken roughly at the time points indicated in Fig. 7a, b.

**Fig. 8:**
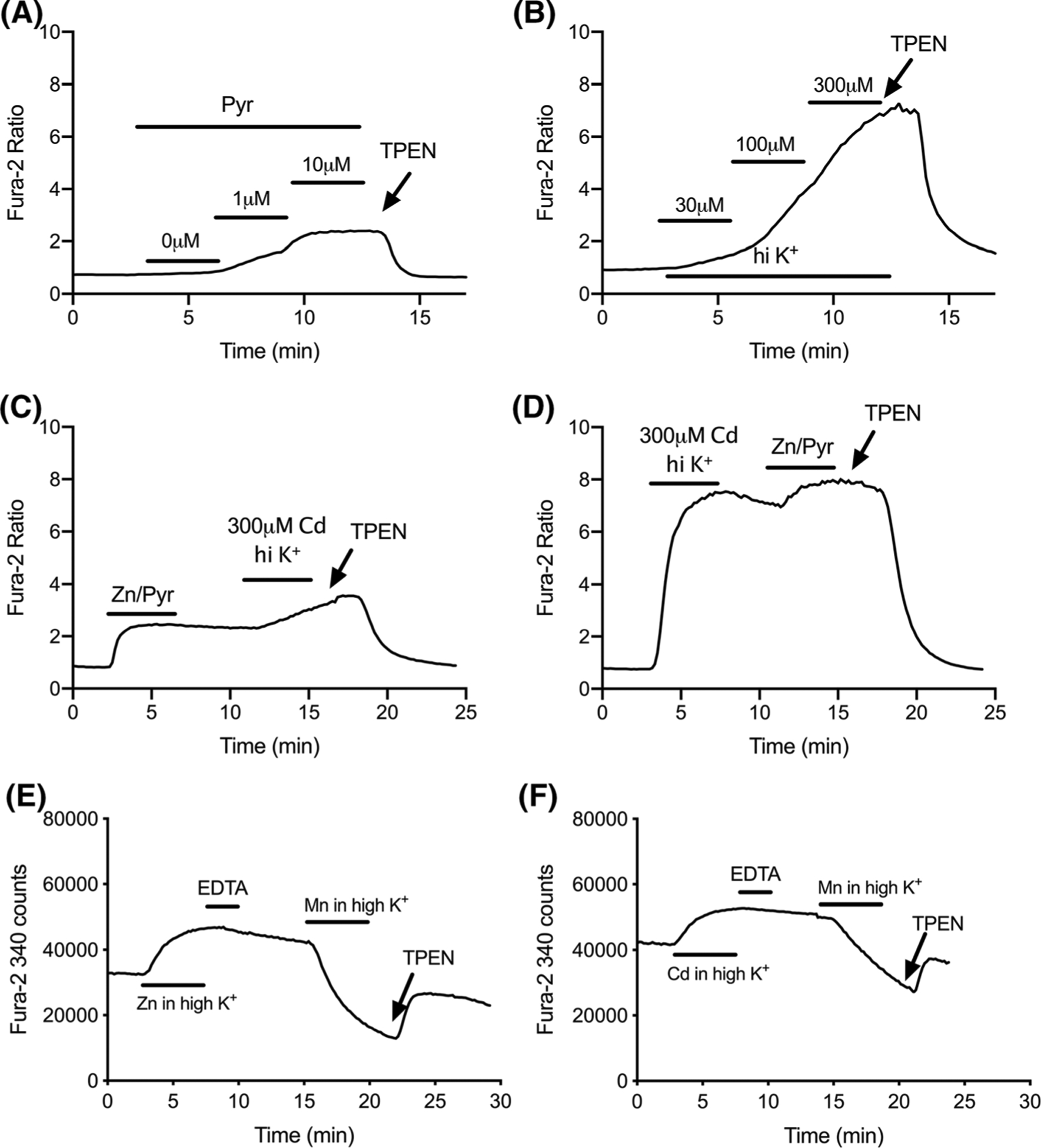
Using fura-2 to distinguish between [Zn^2+^]_i_ and [Cd^2+^]_i_. **a** Representative fura-2 traces recorded from cells treated for 3-min sequential additions of 20 μM sodium pyrithione in the presence of 0, 1 and 10 μM ZnSO_4_, and reversed with 50 μM TPEN. **b** Representative fura-2 traces recorded from cells treated sequentially for 3 min each with 30, 100 and 300 μM CdCl_2_ in the presence of depolarizing buffer, then reversed with 50 μM TPEN. **c** Representative fura-2 trace of cells treated for 5 min with 10 μM ZnSO_4_ and 20 μM sodium pyrithione, followed by 3 min rinse, then 5 min of 300 μM CdCl_2_ in the presence of depolarizing buffer. **d** Representative fura-2 trace from cells treated for 5 min with 300 μM CdCl_2_ in the presence of depolarizing buffer, followed by 3 min rinse then 5 min of 20 μM sodium pyrithione with 10 μM ZnSO_4_. Experiments were terminated with 50 μM TPEN. Fura-2 counts at 340 nm from cells treated with either 50 μM ZnSO_4_ (**e**) or CdCl_2_ (**f**) in high KCl buffer for 5 min, followed by 50 μM EDTA, then rinsed with regular buffer, treated with 50 μM MnCl_2_ in high KCl buffer, and finally reversed with 50 μM TPEN

The abundance of intracellular Zn^2+^ binding sites that also have high affinity for Cd^2+^ raises the possibility of Cd^2+^-induced mobilization of intracellular Zn^2+^. In that scenario, [Zn^2+^]_i_ could contribute importantly to the dye signals we have attributed to [Cd^2+^]_i_. Because there exists no live-cell probe that is sensitive to Cd^2+^ but insensitive to Zn^2+^, this question cannot be answered conclusively. However, in a series of experiments that closely examines the fura-2 responses to [Zn^2+^]_i_ or [Cd^2+^]_i_, our data suggests that Cd^2+^ is the dominant free species inside the cell after depolarization-induced [Cd^2+^]_i_ accumulation, while [Zn^2+^]_i_ makes little if any contribution to the dye signal. In Fig. 8, cells were loaded with Zn^2+^ (0, 1, and 10 μM) using the ionophore sodium pyrithione (Fig. 8a), or with Cd^2+^ (30, 100, and 300 μM) using depolarization (Fig. 8b) in order to achieve a maximum dye response for each metal. For Zn^2+^, the ratio rose by 1.61 ± 0.06 ratio units (Fig. 8a), compared to 6.21 ± 1.01 units for Cd^2+^ (Fig. 8b). This shows that fura-2 can discriminate [Cd^2+^]_i_ from [Zn^2+^]_i_, based on the much greater dynamic range of ratio that is attained with [Cd^2+^]_i_. We then used this difference to determine which metal dominates the dye response in a paradigm where cells are loaded with both metals sequentially. Again using sodium pyrithione, we achieved high [Zn^2+^]_i_, then followed with Cd^2+^ under depolarization conditions (Fig. 8c). The initial Zn^2+^ challenge produced a peak ratio similar to the values obtained in Fig. 8a. The subsequent [Cd^2+^]_i_ load then further elevated the ratio beyond the [Zn^2+^]_i_ plateau. But when the sequence was reversed (Fig. 8d), a [Zn^2+^]_i_ load had little effect on a fura-2 response already dominated by [Cd^2+^]_i_. These data indicate that fura-2 favors [Cd^2+^]_i_ when both metals are present. Lastly, we examined the ability of [Mn^2+^]_i_ to quench fura-2 signals elicited by more modest metal loads (Fig. 8e, f). We hypothesized that, due to fura-2’s significantly higher affinity for Cd^2+^ compared to Zn^2+^ (K_D_ ~10^−12^ vs. ~ 10^−9^M, respectively), the rate of Mn^2+^ quench would be slower for a signal dominated by [Cd^2+^]_i_. Conversely, if the “[Cd^2+^]_i_ response” was actually due to [Zn^2+^]_i_ that was displaced by Cd^2+^, then the quenching kinetics in the Cd^2+^ trial would not differ from the Zn^2+^ trial. Our data show that [Mn^2+^]_i_ extinguished the 340 nm component of the [Cd^2+^]_i_ signal at a rate slower than a corresponding [Zn^2+^]_i_ signal (quenching rate for [Cd^2+^]_i_, in counts per minute, − 8.61 ± 0.64 counts/min; for [Zn^2+^]_i_ − 9.89 ± 0.77), in cells depolarized in the presence of 50 μM Zn^2+^ or Cd^2+^. When considered as a whole, the data in Fig. 8 suggest that the intracellular [Cd^2+^]_i_ loads achieved in our model behave as free Cd^2+^ that interacts directly with the dye. It remains possible that some Zn^2+^ is liberated from intracellular binding sites, but this appears to be minimal.

## Discussion

Our data provide a thorough comparison of probes that might be used for live-cell [Cd^2+^]_i_ detection in models of acute [Cd^2+^]_i_ accumulation. FluoZin-3 demonstrated several strengths in this context: it proved sensitive, it responded strongly across a range of Cd^2+^ concentrations, and its performance was not obviously altered by [Ca^2+^]_i_ changes. This last property lends FluoZin-3 a clear advantage over fura-2. Other convenient features of FluoZin-3 include single wavelength excitation and emission parameters that are common to many “green” probes, so it can be imaged with standard epifluorescence equipment, or with a 488 nm confocal laser. However, FluoZin-3 offers none of the potential benefits of ratiometric imaging, because its spectral maxima do not change with metal binding, and they remain at the same wavelengths regardless of the partnered metal. Also, there is the difficult question of [Zn^2+^]_i_ contribution to the [Cd^2+^]_i_ signal, which we consider at length in the paragraphs further below.

The utility of fura-2 as a [Cd^2+^]_i_ detector was established in a landmark study identifying L-type VGCCs as a potential route of intracellular Cd^2+^ accumulation (Hinkle et al. 1992). Subsequent work used fura-2 to identify Ca^2+^-permeable pathways as routes of Cd^2+^ uptake in various cell types including cerebellar neurons (Usai et al. 1999), lymphoma cells (Li et al. 2000), melanotrophs (Shibuya and Douglas 1992), and pheochromocytoma cells (Hinkle and Osborne 1994). A major limitation common to all those experiments was the need to leave Ca^2+^ out of the extracellular solutions, in order to minimize [Ca^2+^]_i_ contribution to the fura-2 signal. This complication is highlighted in our data, which show a fura-2 response to [Cd^2+^]_i_ that is progressively distorted as higher amounts of Ca^2+^ are added back into the extracellular solution. At physiological extracellular Ca^2+^ (1.4 mM), the resulting [Ca^2+^]_i_ load clearly becomes an important component of the fura-2 signal. Because fura-2 affinity for Cd^2+^ (K_D_ ~ 10^−12^M) is about five orders of magnitude higher than for Ca^2+^ (K_D_ ~10^−7^M) the ability of [Ca^2+^]_i_ to interfere with the [Cd^2+^]_i_ response is perhaps counterintuitive. However, here it is necessary to remember that methods using passive dye loading of AM esters can achieve very high intracellular dye concentrations— hundreds of micromolar, possibly even millimolar. In such conditions, the binding reaction is more a function of reactant concentrations, while the K_D_ becomes less important (Dineley et al. 2002). This explains in part how a large [Ca^2+^]_i_ burden might occupy a significant fraction of the available fura-2, in the presence of a much smaller amount of [Cd^2+^]_i_. The difficulty in distinguishing [Cd^2+^]_i_ amidst [Ca^2+^]_i_ is further complicated by the fact that the emission profile for both metals is essentially the same, making it impossible to resolve [Ca^2+^]_i_ from [Cd^2+^]_i_ based on the fura-2 emission maximum, isosbestic point, or other spectral properties. Nevertheless, fura-2 can be an effective live cell [Cd^2+^]_i_ sensor in circumstances where measures are taken to limit [Ca^2+^]_i_ changes. Furthermore, and in contrast to FluoZin-3, the ratiometric capabilities of fura-2 afford some protection against signal artifacts that arise from variable dye loading, photobleaching, and focus drift, all of which complicate the use of single wavelength probes, particularly in applications where the change from baseline is expected to be small.

The abundance of intracellular Zn^2+^ binding proteins that also possess high affinity for Cd^2+^ raises the possibility that [Zn^2+^]_i_ that is displaced by Cd^2+^ contributes importantly to the dye signals that we have attributed to [Cd^2+^]_i_. With respect to fura-2, we presented several lines of evidence (Fig. 8) suggesting that [Cd^2+^]_i_ is the major binding partner. The dynamic range of fura-2 proved to be several times greater for [Cd^2+^]_i_ compared to [Zn^2+^]_i_, and we used that difference in apparent efficacies to reveal that [Cd^2+^]_i_ outcompetes [Zn^2+^]_i_ in circumstances where both metals are abundant. Furthermore, in trials involving more modest metal accumulation, Mn^2+^ quenching of fura-2 was slower after [Cd^2+^]_i_, compared to [Zn^2+^]_i_. The simplest interpretation of these data suggests that Cd^2+^ is the major free species, while liberated Zn^2+^ appears to be minimal. Yet it remains possible that substantial [Zn^2+^]_i_ is mobilized and furthermore that it might be better reflected in the FluoZin-3 traces. Unfortunately, there is no practical way to discern any [Zn^2+^]_i_ component within a [Cd^2+^]_i_-induced FluoZin-3 signal. The fixed spectral character of FluoZin-3 does not permit nuanced interrogation with different metals, and FluoZin-3 has no good quenching partners in live cell applications. Cu^2+^ is the only potent and effective quencher of FluoZin-3 (Zhao et al. 2009), but it is not useful in this context due to its inherent redox lability and poor membrane permeation. Finally, the viability of the quenching approach with fura-2 is facilitated by its extremely high Cd^2+^ affinity, which exceeds the Zn^2+^ affinity by three orders of magnitude. Whereas with FluoZin-3, Zn^2+^ and Cd^2+^ affinities are separated by a factor of only 10–30 fold—an actually modest difference that would be hard to practically exploit in live-cell imaging. The upshot is that no trick exists for discerning a [Zn^2+^]_i_ contribution to a FluoZin-3 response triggered by [Cd^2+^]_i_. A more informative consideration of the general question of Cd^2+^-induced release of intracellular Zn^2+^ awaits the invention of a live-cell probe that is sensitive to one of these metals but not the other.

The combination of fura-2 and FluoZin-3 offers a potentially useful approach for studying the relationship between [Cd^2+^]_i_ and [Ca^2+^]_i_. Again, considering only their respective affinities it seems surprising that FluoZin-3 (K_D_ Cd^2+^ ~ 10^−7^ M), could effectively compete for Cd^2+^ in the presence of fura-2 (K_D_ Cd^2+^ ~ 10^−12^ M). This seeming paradox is perhaps explained by competitive binding dynamics, where a surfeit of [Ca^2+^]_i_ consumes much of the fura-2 binding capacity, leaving [Cd^2+^]_i_ free to bind with FluoZin-3.

In the current study, we were most surprised by the nearly complete failure of Leadmium Green to respond to [Cd^2+^]_i_. Other groups have used Leadmium Green to report Cd^2+^ uptake in various plant and animal systems (Miura et al. 2013; Greger et al. 2016; Johnson et al. 2018, 2019). Our own prior work with a mouse hippocampal cell line concluded that Leadmium Green is a reliable [Cd^2+^]_i_ sensor, so long as any concomitant [Zn^2+^]_i_ perturbations are kept minimal (Malaiyandi et al. 2016). However, Leadmium Green did not respond to depolarization-induced [Cd^2+^]_i_ accumulation in the MIN6 cells used in the present study. The inconsistency between our current and previous data might be explained by the different methods used to deliver [Cd^2+^]_i_: the previous work used the ionophore pyrithione, which probably introduces a much larger [Cd^2+^]_i_ burden compared to what can be achieved via activation of endogenous ion channels. Other probes we evaluated, e.g. indo-1, Rhod-5N, mag-fura-2, etc., had shown promise in cell-free systems (Vo-Dinh et al. 1994; Hyrc et al. 2000; Soibinet et al. 2008; Johnson 2010). But when applied to our MIN6 cell model of [Cd^2+^]_i_ accumulation, none of them proved effective as live cell [Cd^2+^]_i_ sensors. These results underscore the inherent difficulties in translating in vitro Cd^2+^ responsiveness into practical utility inside the living cell.

## Conclusion

In this study we have carefully evaluated a collection of commercially available Cd^2+-^ sensitive fluorophores for their utility as [Cd^2+^]_i_ probes. FluoZin-3 and fura-2 proved most effective, but care must be taken to assure that their signals are not confounded by [Zn^2+^]_i_ or [Ca^2+^]_i_, respectively. The combination of FluoZin-3 and fura-2 offers a novel method for the simultaneous detection of [Cd^2+^]_i_ and [Ca^2+^]_i_.
